# Association between severity of periodontitis and clinical activity in rheumatoid arthritis patients: a case–control study

**DOI:** 10.1186/s13075-019-1808-z

**Published:** 2019-01-18

**Authors:** Beatriz Rodríguez-Lozano, Jerián González-Febles, Jorge Luis Garnier-Rodríguez, Shashi Dadlani, Sagrario Bustabad-Reyes, Mariano Sanz, Fernando Sánchez-Alonso, Carlos Sánchez-Piedra, Enrique González-Dávila, Federico Díaz-González

**Affiliations:** 1 Servicio de Reumatología, Hospital Universitario de Canarias, La Laguna, Spain; 2 Periodoncia, Facultad de Odontología, Universidad Complutense, Madrid, Spain; 3 Clínica Dental Dr. Garnier, S/C de Tenerife, Spain; 4 Grupo de Investigación de Etiología y Tratamiento de las Enfermedades Periodontales (ETEP), Facultad de Odontología, Universidad Complutense, Madrid, Spain; 5 Unidad de Investigación de la Sociedad Española de Reumatología, Madrid, Spain; 6 Departamento de Estadística e Investigación Operativa, Universidad de La Laguna, S/C de Tenerife, La Laguna, Spain; 7 Departamento de Medicina, Facultad de Medicina, Calle Ofra s/n 38320, La Laguna, Spain

**Keywords:** Rheumatoid arthritis, Periodontitis, Disease activity

## Abstract

**Background:**

A high prevalence of periodontitis has been reported in rheumatoid arthritis (RA) patients, although the strength of this association, its temporal link and the possible relationship between the severity of periodontitis and RA disease activity remain unclear. The objective of this work was to investigate whether periodontitis is associated with RA and whether periodontitis severity is linked to RA disease activity.

**Methods:**

This case–control study included 187 patients diagnosed with RA and 157 control patients without inflammatory joint disease. RA disease activity and severity were evaluated by the Disease Activity Score 28, the Simplified Disease Activity Index, the Clinical Disease Activity Index, rheumatoid factor, anti-citrullinated protein antibody titers, the erythrocyte sedimentation rate, C-reactive protein, presence of extra-articular manifestations and type of RA therapy. Exposure severity was assessed by the following periodontal parameters: plaque index, bleeding on probing, probing pocket depth and clinical attachment levels. Sociodemographic variables and comorbidities were evaluated as confounding variables. Outcome and exposure variables were compared by both parametric and nonparametric tests, and possible associations were assessed through regression analysis with a calculation for the adjusted odds ratio (OR).

**Results:**

A significant association was demonstrated between periodontitis and RA with an adjusted OR of 20.57 (95% CI 6.02–70.27, *p* < 0.001). Compared with controls, all parameters related to periodontal status (plaque index, bleeding on probing, probing pocket depth and clinical attachment levels) were significantly worse in RA patients (*p* < 0.001). Periodontitis severity was significantly associated with RA disease activity (*p* < 0.001), showing in an ordinal logistic regression model an association between periodontal severity and disease activity with an adjusted OR of 2.66 (95% CI 1.24–5.74, *p* = 0.012).

**Conclusion:**

A significant association was demonstrated between periodontitis and RA, independent of other confounders. This association was more evident in patients with pronounced periodontal disease and higher RA disease activity.

**Electronic supplementary material:**

The online version of this article (10.1186/s13075-019-1808-z) contains supplementary material, which is available to authorized users.

## Background

Rheumatoid arthritis (RA) is a systemic autoimmune chronic disease that causes inflammation and the proliferation of synovial membranes in di-arthrodial joints. When uncontrolled, RA destroys articular structures, resulting in functional disability, decreased quality of life and reduced life expectancy, mainly due to an increased risk of cardiovascular events [1]. RA is a multifactorial disease of unknown etiology in which genetic susceptibility, environmental and hormonal factors interact in complex ways [2]. The current understanding of RA pathogenesis postulates that the activity of peptidil arginine deiminase (PAD), an enzyme that transforms arginine into citrulline, which causes a posttranslational modification in structural proteins, could be the main event in the early stages of RA. Although protein citrullination is not exclusive to RA, the formation of anti-citrullinated protein antibodies (ACPA) is almost unique to this disease, these antibodies being a marker of aggressive RA [3, 4].

Periodontitis is a chronic inflammatory disease characterized by the destruction of the periodontal ligament and alveolar bone, which if untreated can lead to tooth loss. Periodontitis is considered one of the most prevalent chronic inflammatory noncommunicable diseases [5, 6]. Recent epidemiological studies have highlighted the important links between periodontitis and other chronic inflammatory diseases such as diabetes or cardiovascular disease [7, 8]. In this regard, clinical [9, 10] and epidemiological studies [11] have reported a high prevalence of periodontitis and tooth loss in RA patients, although the strength of this association, its temporal link and the possible relationship between the severity of periodontitis and RA disease activity remain unclear. Mikuls et al. [9] reported a statistically significant association between periodontitis and swollen joints, high-level disease activity and serum levels of ACPA in patients with RA. Other studies, however, were unable to find this link [12]. There is evidence that periodontitis and RA share genetic risk factors such as the HLA-DRB1 allele of the MHC class II molecules [13]. Similarly, environmental risk factors, such as smoking, socioeconomic status and obesity, may influence both diseases [2]. In spite of these well-established shared risk factors, some authors have explained this association through the activity of some periodontal pathogens [2]. Specifically, *Porphyromonas gingivalis*, a Gram-negative, anaerobic bacterium, by releasing a specific deaminase is able to induce protein citrullination, which through a process of mimicry might stimulate ACPA formation in RA patients [9, 14]. In severe periodontitis, the chronic exposure of citrullinated proteins and the subsequent development of autoantibodies could explain this reported association between periodontitis and RA, but the existing evidence is still limited [15, 16].

The objective of this case–control study was to investigate the link between RA and periodontitis, to assess whether RA disease activity is associated with periodontitis severity and to determine the degree to which this association is affected by shared risk factors.

## Patients and methods

### Study population

RA patients (cases) were consecutively included from those outpatients attending the Department of Rheumatology of the Hospital Universitario de Canarias in Tenerife from January to September 2016 following a design stratified by the use of biologics (see later). RA patients aged 18 years and older fulfilling the 2010 ACR/EULAR classification criteria [17] were included and categorized as early-onset RA (≤ 2 years of disease evolution) and established RA (> 2 years of disease evolution). Controls were those patients matching in age suffering from osteoarthritis or soft tissue rheumatic diseases seen correlatively in the rheumatology outpatient clinic of our institution during the same time period. Patients having fewer than eight teeth [12], having received periodontal or antibiotic treatment during the previous 6 months [18], with joint replacement(s), in need of antibiotic prophylaxis, or being treated with cyclosporine A or anticonvulsants [19] were excluded.

All enrolled subjects were informed about the objectives and characteristics of this study and signed a written informed consent previously approved by the ethics committee of Hospital Universitario de Canarias.

### Study variables

#### Outcome and exposure variables

For all RA patients the following parameters were recorded: 28-joint Disease Activity Score using the erythrocyte sedimentation rate (ESR) (DAS28) or C-reactive protein (DAS28-CRP) [20], the Simplified Disease Activity Index (SDAI) [21], the Clinical Disease Activity Index (CDAI) [22], the Health Assessment Questionnaire (HAQ) [23], rheumatoid factor (RF), ACPA and extra-articular manifestations. Patients were categorized into remission, low, moderate and high disease activity when at least two of the DAS28, DAS28-CRP and SDAI agreed on the level of disease activity (combined index). Furthermore, in patients treated with biologics, the RA activity was also expressed as the mean ± SD of DAS28-CRP values obtained during the 2-year period prior to the periodontal evaluation.

Patients’ periodontal status was evaluated for cases and controls using the following parameters (see Additional file 1): full mouth plaque index (PI) [24], bleeding on probing (BoP) [25], probing pocket depth (PPD), recession (REC), clinical attachment level (CAL) and tooth loss. Based on these parameters, patients were categorized into one of three case definitions of periodontitis [26]: Level 0, individuals with a healthy periodontium and up to one proximal site with loss of attachment ≥ 3 mm; Level 1, presence of proximal attachment and loss ≥ 3 mm in ≥ 2 nonadjacent teeth; and Level 2, presence of proximal attachment loss ≥ 5 mm in ≥ 30% of teeth. PI and BoP were also recorded as percentages. In addition, when calculating the number of teeth, dental implants and third molars were excluded.

The presence of comorbidities, such as diabetes mellitus, osteoporosis, myocardial infarction or dyslipidemia, as well as body mass index (BMI) were recorded for all patients. In RA patients, any history of therapy with glucocorticoids, synthetic disease-modifying antirheumatic drugs (sDMARDs) and bDMARDs was also recorded.

Anthropometric and socioeconomic variables were also recorded including BMI, smoking status, stress by the Perceived Stress Scale (PSS-14) categorized into high stress yes (> 28 points) or no (≤ 28 points) [27, 28] and social welfare indicators using the Graffar Scale questionnaire [29].

### Study design

This was an observational, case–control study of RA patients treated in a single rheumatology department who were assessed for the presence and severity of periodontal disease.

#### Medical examination

Cases were subjected to a routine medical examination. The patient’s and care provider’s global assessments of disease activity were based on a 100-mm visual analog scale (VAS), and the RA functional index was assessed by the HAQ. Disease activity was calculated by means of DAS28, DAS28-CRP, SDAI and CDAI scores, and the medications used were logged from the patient files and by asking the patient during medical examination. Blood samples were tested for plasma RF and CRP using an immunoturbidimetric assay (Roche/cobas® 8000 Modular Analyzer Series; Roche Diagnostics, USA) and for ACPA (anti-cyclic citrullinated peptide anti-CCP-2 by Immunoscan CCPlus®; Euro Diagnostica), with a positive value established as that exceeding 25 U/ml in both serological tests and 3 mg/l in the CRP test.

#### Periodontal examination

A full oral and periodontal examination was carried out by two experienced periodontists masked to the patient’s diagnosis. A kappa test showed 70% inter-examiner concordance. Queried dental visits and symptoms of oral sicca were also recorded.

Full mouth PPD and CAL measurements were registered using an UNC-15 periodontal probe (six sites per tooth), excluding third molars and implants. Full mouth periapical X-ray scans were taken to confirm the periodontal diagnosis when it was not clear. Although subjects were informed on their periodontal status and advised to seek periodontal therapy when appropriate, no periodontal therapy was rendered as part of this investigation.

### Sample size and statistical analyses

For sample size calculation we used the DAS28 score as the main outcome variable, with the assumption of the difference between RA patients exposed (with periodontitis) versus nonexposed (without periodontitis) reported by Mikuls et al. [9]. A sample size of 166 patients was estimated to provide 80% power to achieve statistical significance at a two-sided significance level of 0.05, and assuming a 15% dropout rate, and therefore we included 190 patients per group. Thirty percent of RA patients in our service were being treated with biologic disease-modifying antirheumatic drugs (bDMARDs). To maintain this proportion, 56 biologic-treated patients were included in our study. Descriptive statistics were presented as means, standard deviations (SDs) and frequency distributions. Inter-group comparisons for the continuous variables were studied with a *t* test and one-way ANOVA with Dunnett’s post test. The Mann–Whitney *U* test and Kruskall–Wallis test were used for nonparametric continuous variables and a chi-squared test for categorical variables, using Fisher’s exact test in the comparison of 2 × 2 tables with expected values < 5. The degree of relationship between the categorical ordinal variables was measured with Kendall’s tau-b correlation coefficient.

The study of the relationship between periodontitis (exposure) and RA (outcome) was carried out with a logistic regression model examining the odds ratio (OR) and 95% confidence intervals (CIs). Additionally, these values were adjusted for possible confounders (covariates) such as age, sex, sociodemographic index, annual dental prophylaxis, tobacco use, BMI and comorbidities. In RA patients, the relationship between periodontitis severity (classified as Level 0 + 1 and Level 2) and RA disease activity levels (classified as remission, low and moderate + high) was studied with an ordinal logistic regression model taking into account the information from the previous covariates. The coefficients of the different covariates and factors verified the test of parallel lines (*p* = 0.340). The linear predictor of cumulated probabilities was assessed, maintaining the sign of the thresholds and changing the sign of coefficient estimations of the different covariates [30]. A linear regression analysis was performed to elucidate any association between the DAS28 and DAS28-CRP scores and periodontal attachment loss. Finally, the relationships between periodontitis levels and RA treatments (one or more DMARDs, biologics and steroids), and positivity for autoantibodies (RF and anti-CCP), were analyzed by chi-squared test.

The statistical analyses were performed using the Stata statistical package (Stata Statistical Software version 13.1; StataCorp LP, College Station, TX, USA).

## Results

### Characteristics of RA patients and controls

In total, 380 patients were screened. Three RA patients and 12 controls decided not to participate after reading the informed consent. Twenty-one controls did not attend their appointments for periodontal evaluations. Table 1 presents the demographic characteristics of the 344 patients recruited: 187 cases (RA patients) and 157 controls (osteoarthritis and soft tissue rheumatic disease). When compared with controls, RA patients were significantly different with regard to gender, socioeconomic status (lower levels in RA patients), smoking habit (with a higher number of current and former smokers in the RA group than in the control group), dyslipidemia (hypertriglyceridemia) and osteoporosis. Both groups were similar in age, BMI, diabetes mellitus, cardiovascular disease and stress level.

**Table 1:** Anthropometric and sociodemographic characteristics and comorbidities in RA patients and controls

	RA patients		Controls		*p*
	*N*	%	*N*	%	
Gender					
Female	147	78.61	101	64.33	
Male	40	21.39	56	35.67	
Ratio female/male	3.6		1.8		0.003*
Mean (SD) age (years)	54.4 (10.8)		55.5 (23.7)		
Race					
Caucasian	182	97.33	152	96.81	
Diagnosis					
Osteoarthritis	–	–	118	75.15	
Back pain	–	–	23	14.65	
Scapulohumeral peri-arthritis	–	–	10	6.36	
Others	–	–	6	3.84	
Graffar Scale					
High	13	6.95	25	15.92	
Medium	34	18.18	48	30.57	
Low	68	36.36	55	35.03	
Relative poverty	63	33.69	28	17.83	
Extreme poverty	8	4.28	0	0	< 0.001*
Annual dental prophylaxis	80	43.01	90	57.32	0.008*
Mean (SD) ESR (mm/h)	26.03 (16.8)		16.97 (12.92)		
Periodontally healthy (Level 0)	21.80 (7.46)		16.06 (8.98)		
Periodontitis (Level 1 + 2)	25.86 (16.68)		19.05 (15.40)		0.022**
Mean (SD) CRP (mg/L)	5.14 (7.00)		2.85 (4.03)		
Periodontally healthy (Level 0)	4.43 (2.34)		1.72 (1.08)		
Periodontitis (Level 1 + 2)	4.86 (5.21)		3.26 (4.83)		0.069**
Bone mineral density					
Normal	64	44.14	113	80.14	
Osteopenia	47	32.41	16	11.35	
Osteoporosis	34	23.45	11	7.8	< 0.001*
Tobacco					
Never	105	56.15	125	79.62	
Current	36	19.25	14	8.92	
Former smoker	46	24.6	18	11.46	< 0.001*
Stress	50	27.03	46	29.30	
Diabetes	23	12.3	13	8.33	
Type II	19	10.16	12	7.64	
Dyslipidemia	100	53.48	71	45.22	
Hypercholesterolemia	51	27.27	56	35.67	
Hypertriglyceridemia	21	11.23	7	4.46	
Mixed hyperlipidemia	28	14.97	8	5.1	< 0.001*
Hypertension	58	31.02	35	22.29	
Myocardial infarction	8	4.28	3	1.92	
Mean (SD) BMI (kg/m^2^)	27 (4.7)		27.9 (4.4)		
Normal, 18.5–24.99	52	27.81	47	30.13	
Overweight, 25–29.99	79	42.25	71	45.51	
Obesity I, 30–34.99	46	24.6	28	17.95	

Data represent number, percentage or mean (SD). *p* > 0.05 not shown

*BMI* body mass index, *CRP* C-reactive protein, *ESR* erythrocyte sedimentation rate, *RA* rheumatoid arthritis, *SD* standard deviation

*Inter-group comparisons

**Intra-group comparisons

The clinical characteristics of RA patients are summarized in Table 2. Of the 187 RA cases, 78.6% were female, the mean age was 54.4 ± 10.8 years and the mean disease follow-up was 8.8 ± 7.32 years. Thirty-five patients (18.72%) had early RA (ERA). ACPAs were detected in 114 patients (67.9%) while 138 patients (74.2%) were RF positive. Mean ± SD disease activity, as assessed by the different indexes used, was: DAS28, 3.81 ± 1.31; DAS28-CRP, 3.18 ± 1.18; SDAI, 14.49 ± 10.74; and CDAI, 12.68 ± 10.19. Based on disease duration, we observed a higher proportion of patients with high activity in ERA patients (31.43%) compared to established RA (9.21%) (*p* = 0.001) (see Additional file 2: Table S1).

**Table 2:** Clinical characteristics, disease activity and treatment of RA patients

	*N*	%
Gender		
Female	147	78.61
Male	40	21.39
Mean (SD) age (years)	54.4 (10.8)	
Mean (SD) time evolution (years)	8.88 (7.32)	
Early RA	35	18.72
Established RA	152	81.28
Rheumatoid factor		
Seropositive	138	74.19
Rheumatoid factor concentration (IU/ml)		
Low (≤ 90)	49	36.84
Moderate (91–300)	56	42.11
High (> 300)	28	21.05
Anti-CCP		
Positive	114	67.86
Anti-CCP concentration (U/ml)		
Low (≤ 75)	30	27.27
Moderate (76–300)	43	39.09
High (> 300)	37	33.64
Level of activity, mean (SD)		
DAS28	3.81(1.31)	
DAS28-CRP	3.18(1.18)	
SDAI	14.49(10.74)	
CDAI	12.68(10.19)	
Disease activity (combined index)		
Remission	38	20.32
Low	39	20.90
Moderate	85	45.45
High	25	13.43
HAQ	0.760 (0.62)	
Glucocorticoid therapy		
No glucocorticoids	99	52.94
Glucocorticoids	88	47.06
Current dosage GC, mean (SD) (mg/day)	2.85 (4.24)	
Low < 7.5	65	73.86
Moderate 7.5–20	22	25
High > 20	1	1.14
Type of RA therapy		
No treatment	10	5.35
sDMARDs	99	52.94
≥ 2 sDMARDs	22	11.76
bDMARDs	56	29.95

Data represent number, percentage or mean (SD)

*anti-CCP* anti-cyclic citrullinated peptide, *bDMARD* biologic disease-modifying antirheumatic drug, *CDAI* Clinical Disease Activity Index, *DAS28* 28-joint Disease Activity Score with erythrocyte sedimentation rate, *DAS28-CRP* 28-joint Disease Activity Score with C-reactive protein, *GC* corticosteroids, *HAQ* Health Assessment Questionnaire, *RA* rheumatoid arthritis, *SD* standard deviation, *SDAI* Simplified Disease Activity Index, *sDMARD* synthetic disease-modifying antirheumatic drug

Ninety-nine patients (52.94%) received sDMARD as monotherapy, mainly methotrexate (79.14%), while only 12.3% of patients received two or more sDMARDs (9.6% methotrexate and leflunomide). The remaining 56 patients (29.95%) were treated with a bDMARD. Almost half of RA patients (*n* = 88, 47.06%) received steroids with a mean daily dose of 5.47 ± 4.59 mg of prednisone, for a mean time of 3.79 ± 5.09 years.

### Prevalence and severity of periodontitis in RA and control patients

Different degrees of periodontitis (Level 1 + 2) were observed in 97.33% of RA patients and 66.24% of patients in the control group (*p* < 0.001). Severe periodontitis (Level 2) was significantly higher in RA patients (44.92%) than in controls (12.1%) (*p* < 0.001) (Table 3). All parameters related to periodontal status (plaque indices, BOP scores, PPDs, number and percentages of pockets ≥ 5 mm, mean levels of CA loss and tooth loss) were significantly worse in RA (Table 3).

**Table 3:** Prevalence and severity of periodontitis in RA patients and controls

	RA patients			Controls			*p*
	*N*	%	Mean (SD)	*N*	%	Mean (SD)	
Periodontitis^a^							
Level 0	5	2.67		53	33.76		< 0.001
Level 1	98	52.41		85	54.14		
Level 2	84	44.92		19	12.1		< 0.001
Level 1 + 2	182	97.33		104	66.24		< 0.001
Periodontal variables							
Mean PI			0.74 (0.48)			0.56 (0.40)	< 0.001
Mean PPD			3.08 (0.63)			2.69 (0.48)	< 0.001
CAL			3.99(1.27)			3.34 (1.03)	< 0.001
Tooth loss			6.31 (5.28)			4.01 (4.48)	< 0.001
N° PPD ≥ 5 mm			14.60 (17.03)			6.37 (11.50)	< 0.001
% PPD ≥ 5 mm			0.12 (0.14)			0.05 (0.08)	< 0.001
% BoP			0.63 (0.23)			0.42 (0.26)	< 0.001

Data represent number, percentage or mean (SD)

*% BoP* percentage of sites with bleeding on probing, *CAL* clinical attachment level, *%PPD ≥ 5 mm* percentage of pockets ≥ 5 mm, *N° PPD ≥ 5 mm* number of pockets ≥ 5 mm, *PI* plaque index, *PPD* probing pocket depth, *RA* rheumatoid arthritis, *SD* standard deviation, *Tooth loss* number of missing teeth

^a^Level 1, periodontitis; Level 2, periodontitis according to Tonetti’s classification [26]

Table 4 presents the association between RA and periodontitis (Level 1 + 2) with respect to controls with a raw OR of 14.75 (95% CI 5.66–34.4, *p* < 0.001). After adjusting for possible confounders such as age, gender, socioeconomic status, annual prophylaxis, osteoporosis, smoking habit, stress, BMI, hypertension and myocardial infarction, the multivariable analysis shows an adjusted OR of 20.57 (95% CI 6.02–70.27, *p* < 0.001). When this association was studied with respect to periodontitis Level 1 or Level 2, the adjusted OR was 14.78 (95% CI 4.23–51.68) and 81.01 (95% CI 18.59–353.07), respectively. As is shown in Table 4, in our study, male gender, high socioeconomic status and the performance of an annual dental prophylaxis were protective factors of RA. However, the presence of osteoporosis and a tobacco habit, specifically in former smokers, were factors associated with RA.

**Table 4:** Associated factors with RA versus controls: bivariate and multivariate analyses considering periodontitis Level 1, Level 2 or Level 1 + 2

Factor	Bivariate			Multivariate			Multivariate Level 1 + 2		
	OR	95% CI	*p*	Adjusted OR	95% CI	*p*	Adjusted OR	95% CI	*p*
Periodontitis (ref. to no)									
Level 1	12.22	(4.67–31.98)	< 0.001	14.78	(4.23–51.68)	< 0.001			
Level 2	46.86	(16.51–133)	< 0.001	81.01	(18.59–353.07)	< 0.001			
Level 1 + 2	14.75	(5.66–34.4)	< 0.001				20.57	(6.02–70.27)	< 0.001
Age	1.00	(0.98–1.01)	0.564	0.95	(0.92–0.98)	0.003	0.96	(0.93–0.99)	0.003
Gender (ref. to woman)	0.491	(0.30–0.79)	0.004	0.18	(0.07–0.44)	< 0.001	0.23	(0.10–0.54)	0.001
Socioeconomic status (ref. to poverty(Ext + Rel))									
High	0.20	(0.09–0.46)	< 0.001	0.18	(0.05–0.60)	0.006	0.16	(0.05–0.53)	0.003
Medium	0.28	(0.15–0.52)	< 0.001	0.16	(0.05–0.47)	0.001	0.16	(0.05–0.46)	0.001
Low	0.49	(0.28–0.86)	0.012	0.31	(0.13–0.75)	0.010	0.33	(0.14–0.77)	0.011
Annual dental prophylaxis (ref. to no)	0.56	(0.37–0.86)	0.008	0.52	(0.26–1.05)	0.070	0.49	(0.25–0.96)	0.039
Osteoporosis (ref. to no)									
Osteopenia	5.19	(2.72–9.88)	< 0.001	5.95	(2.29–15.42)	< 0.001	5.26	(2.18–12.65)	< 0.001
Osteoporosis	5.46	(2.59–11.50)	< 0.001	8.00	(2.79–22.86)	< 0.001	6.91	(2.53–18.90)	< 0.001
Smoking (ref. to no)									
Smoker	3.06	(1.57–5.98)	0.001	1.08	(0.44–2.65)	0.858	1.62	(0.69–3.81)	0.269
Former smoker	3.04	(1.66–5.56)	< 0.001	7.67	(2.73–21.57)	< 0.001	8.03	(2.93–22.02)	< 0.001
Stress (ref. to no)	0.89	(0.56–1.43)	0.641	1.14	(0.51–2.57)	0.747	1.16	(0.54–2.49)	0.707
BMI (continuous)	1.00	(0.95–1.04)	0.879	0.93	(0.86–1.01)	0.078	0.94	(0.87–1.01)	0.091
HTA (ref. to no)	1.57	(0.96–2.55)	0.071	3.26	(1.35–7.87)	0.008	2.85	(1.26–6.49)	0.012
MI (ref. to no)	2.28	(0.59–8.74)	0.230	2.63	(0.38–18.42)	0.330	2.84	(0.40–20.24)	0.298

*BMI* body mass index, *CI* confidence interval, *Ext + Rel* extreme poverty plus relative poverty, *HTA* hypertension, *MI* myocardial infarction, *OR* odds ratio, *RA* rheumatoid arthritis, *ref* referred

### Association between periodontitis and clinical activity, severity and treatment in RA patients

**Fig. 1:**
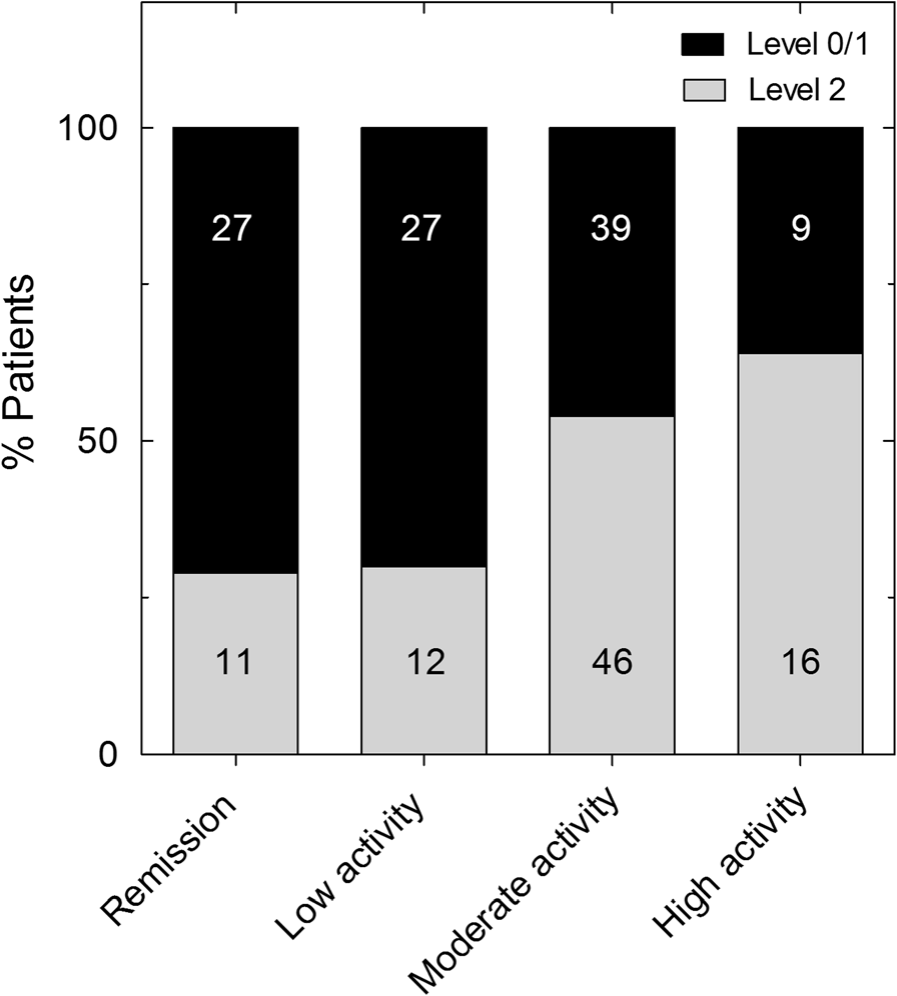
Periodontitis severity in relation to RA clinical activity. Stacked bar graph showing percentage of RA patients presenting no or mild (Level 0/1) and severe (Level 2) periodontitis with respect to RA clinical activity categorized by combined index as remission, low, moderate or high. Numbers in columns represent number of patients in each situation. *p* = 0.0026 by chi-squared test, Kendall’s tau-b = 0.209

Of the RA patients with high disease activity, 64% presented severe periodontitis compared with 30% of patients in remission (Fig. 1). When RA patients were categorized by both disease activity (remission, low, moderate and high activity, using the combined test or DAS28, DAS28-PCR, SDAI and CDAI) and periodontitis severity (Level 0 + 1 or Level 2), and arranged in a natural order, a significant direct trend was observed between the periodontitis level and RA disease activity (*p* = 0.002) (Fig. 1 and Additional file 3: Figure S1). There was a statistically significant higher clinical attachment loss, tooth loss and number of pockets with depth ≥ 5 mm in RA patients with moderate–high activity (*p* < 0.001) compared to controls, both in absolute numbers (see Additional file 3: Figure S2A–C) and in percentages (data not shown). Interestingly, RA patients in remission showed fewer pockets with depth ≥ 5 mm than patients with moderate–high disease activity (*p* < 0.05) (see Additional file 3: Figure S2C). With regard to disease duration, severe periodontitis was more prevalent in ERA patients (60%) compared to established RA patients (41.45%) (*p* = 0.043).

Using an ordinal logistic regression model classifying RA patients by disease activity (remission, low and moderate/high), an association was observed between periodontitis severity and RA disease activity with respect to patients in remission, with an OR of 2.66 (95% CI 1.24–5.74, *p* = 0.012), after adjusting for confounding variables (Table 5). This association was not seen in RA patients with low disease activity (adjusted OR 1.05, 95% CI 0.49–2.26, *p* = 0.48). In the linear regression analysis, statistically significant increases of 0.24 and 0.19 in the DAS28 and DAS28-CRP scores, respectively, were found for each millimeter of periodontal attachment loss in RA patients (*p* = 0.002 and *p* = 0.011, respectively, adjusted by age, gender and smoking) (see Additional file 2: Table S2).

**Table 5:** Association between severe periodontitis and RA disease activity (referred to patients in remission): ordinal logistic regression model

	Estimation	SE	*p*	OR (95% CI)
Bivariate model				
Threshold				
Remission	−0.969	0.210		
Low	0.099	0.193		
Periodontitis = Level 2 (ref. to 0 + 1)	1.105	0.308	< 0.001	3.02 (1.65–5.52)
Multivariate model				
Threshold				
Remission	−3.665	1.703		
Low	−2.504	1.687		
Periodontitis = Level 2 (ref. to 0 + 1)	0.979	0.392	0.012	2.66 (1.24–5.74)
Age	−0.042	0.022	0.060	0.96 (0.92–1.00)
Gender = man (ref. to woman)	−1.225	0.520	0.019	0.29 (0.11–0.81)
Socioeconomic status (ref. to poverty (Ext + Rel))				
High	−1.465	0.713	0.040	0.23 (0.06–0.94)
Medium	−0.186	0.587	0.752	0.83 (0.26–2.63)
Low	−0.138	0.431	0.749	0.87 (0.37–2.03)
Annual dental prophylaxis = Yes (ref. to no)	−0.444	0.395	0.260	0.64 (0.30–1.39)
Osteoporosis (ref. to no)				
Osteopenia	0.034	0.446	0.940	1.03 (0.43–2.48)
Osteoporosis	0.403	0.544	0.458	1.50 (0.52–4.35)
Smoking (ref. to no)				
Smoker	0.303	0.537	0.572	1.35 (0.47–3.88)
Former smoker	0.086	0.460	0.851	1.09 (044–2.69)
Stress = Yes (ref. to no)	0.978	0.497	0.049	2.66 (1.00–7.04)
BMI (continuous)	−0.011	0.043	0.800	0.99 (0.91–1.08)
HTA = Yes (ref. to no)	0.808	0.429	0.059	2.24 (0.97–5.20)
MI = Yes (ref. to no)	1.143	0.983	0.245	3.14 (0.46–21.51)

*BMI* body mass index, *CI* confidence interval, *Ext + Rel* extreme poverty plus relative poverty, *HTA* hypertension, *MI* myocardial infarction, *OR* odds ratio, *RA* rheumatoid arthritis, *SE* standard error

No association was observed between the immunological characteristics of RA (presence of RF or anti-CCP) and the presence of periodontitis. The severity of periodontitis in RA patients was not associated with the use of either sDMARDs, bDMARDs or steroids. Interestingly, when we performed a retrospective subanalysis of disease activity for the 2 years prior to the periodontitis evaluation in the 56 RA patients treated with bDMARDs, we observed that those with periodontitis Level 0 or 1 had a significantly lower mean disease activity (as assessed by DAS28-CRP; 3.29 ± 1.16, *n* = 31) than patients with Level 2 periodontitis (3.74 ± 1.23, *n* = 25) (*p* = 0.0046).

In general, no association was found between periodontitis severity and the presence of extra-articular manifestations. Nevertheless, statistically significant differences were observed in patients with severe periodontitis compared to those without or with mild cases vis-à-vis the presence of rheumatoid nodes (*p* = 0.028) (OR 1.54, 95% CI 1.04–2.24) and pleuritis (*p* = 0.025). There was no association between functional disability, as measured by the HAQ score, and the presence of periodontitis in RA patients.

## Discussion

The most important findings of this work can be summarized as follows: there is an independent association between periodontitis and RA; RA patients suffer at a disproportionate rate as well as with more severe periodontal disease than those without a rheumatic inflammatory condition; and RA patients with more severe periodontitis in terms of higher clinical attachment loss, tooth loss and the number and percentage of pocket depths ≥ 5 mm suffered more active rheumatoid disease.

This study confirms a very strong and independent statistical association between RA and the presence of periodontitis. When this association was studied categorizing by periodontal severity as mild (Level 1) and severe (Level 2), consistent adjusted ORs were obtained: 14.78 (95% CI 4.23–51.68, *p* < 0.001) and 81.01 (95% CI 18.59–353.07, *p* < 0.001), respectively. While initial studies did not demonstrate any association between periodontitis and RA [31–33], more recent investigations have revealed a significant relationship between both conditions, although one dependent on factors such as smoking habit and other systemic conditions [10–12, 34–36]. In the multivariate analysis, we found that age, female gender, socioeconomic status and osteoporosis, all common factors in periodontitis and RA in this association, were highly influential [2, 10]. Although tobacco habit is also a common factor in the relationship between periodontitis and RA [37], we only found a strong association between tobacco use and RA in former smokers, although surprisingly this relationship did not reach statistic significance in current smokers. This result can be explained by the low percentage of current smokers, both in patients (19.2%) and particularly in the control group (8.9%).

A high prevalence of severe periodontitis in RA (44.92%) with respect to controls (12.1%) was found, which confirms other studies (44.96–51% vs 24.6–26%) [12, 35]. Using an ordinal regression model, a link between periodontitis and the clinical activity of RA was observed in our study, with a significant association between periodontitis severity and RA disease activity. Although this relationship has been suggested previously [9], several previous reports are not coincident with this finding [12, 34, 38], in most of which the sample sizes were limited and the patient populations were heterogeneous. In this regard, a recent report by Mobini et al. [38] found no association between periodontitis severity and RA disease activity. Nevertheless, the limited sample size, and an imprecise periodontitis case definition, may have influenced the results of this study. Furthermore, we have found a novel and statistically significant linear correlation between ongoing periodontal attachment loss and clinical disease activity.

We additionally observed that there was a higher number of pockets ≥ 5 mm, tooth loss and clinical attachment loss in moderate-to-high activity RA patients, compared to patients in remission and controls. Moreover, a higher prevalence of severe periodontitis was found in ERA patients compared to established RA patients (60% vs 41.45%), which is in agreement with previous reports [39, 40]. This finding could be linked to the fact that a significantly greater percentage of ERA patients experienced high disease activity (31.43%) than those with established RA (9.21%). The retrospective subanalysis of patients receiving bDMARDs showed that a higher disease activity during the 2 years prior to the periodontal assessment was associated with more severe periodontitis. This strengthens the contention that clinical disease activity is associated with worse periodontal health in RA patients. The potential impact of treatment on periodontal status in disease activity of RA patients has not been well characterized to date.

Our data support that the severity of periodontitis in RA patients is not related to the use of sDMARDs, bDMARDs or steroids. In this regard, previous studies have revealed discrepancies concerning the effects of bDMARDs on the severity of periodontitis in RA patients [12, 39, 41–46]. The reason for this is unclear, but differences in the design of the studies, as well as in periodontitis case definitions, may be responsible for this disagreement. Recently, Ziebolz et al. [46] reported a significant association between periodontal inflammation and RA medication in RA patients with similar disease activity. Therefore, better designed studies, with proper periodontitis case definitions, are needed to elucidate the plausible impact of RA medication on periodontal status.

We have found no relationship between periodontitis and the presence of RF in RA patients. Although there is evidence suggesting a possible association between RF and periodontitis [9, 47], our work is in agreement with most of the studies on the absence of a relationship between periodontitis and positivity for RF [14, 36, 48, 49]. With respect to ACPA, most studies have shown a relationship between periodontitis and the presence of such autoantibodies both in individuals with RA [15, 49] and without RA [50]. In our study, we analyzed the relationship between periodontitis and the presence of ACPA, specifically anti-CCP in RA patients. In terms of seropositivity for anti-CCP, our study did not support this association, which is in agreement with previous studies [15, 47, 49].

Interestingly, we observed a not previously described statistical significant association between severe periodontitis and the presence of rheumatoid nodes. This fact could be explained by the higher frequency of these complications in uncontrolled high-activity RA patients.

There are both some limitations and strong points to this study. The selection of a degenerative articular pathology as a control group may have impacted the results, although other studies have also selected similar controls [9, 12]. There were some differences between RA patients and controls in terms of gender, smoking, socioeconomic status, annual dental prophylaxis and/or osteoporosis. Although such covariates were adjusted in the multivariate analysis, the potential impact of those baseline differences on the final conclusion cannot be ruled out. Moreover, our study design did not address causality and this limitation allows for the interpretation of these data, either as a measure of the severity of the impact of periodontitis on RA disease activity or, conversely, as evidence that RA activity influences the severity of periodontitis. With regard to the statistical power and selection bias, a large sample size from the target population in different stages of disease progression and activity was recruited, which reduced the selection bias. We adopted the case definition of periodontitis recommended by the European Federation of Periodontology [26], which has been extensively used in epidemiological studies [51–53] and has proven its validity. We conducted full mouth examinations, which differs from other similar studies that made only partial mouth evaluations. The assessment was carried by two experienced periodontists and showed high inter-examiner reproducibility. This may have resulted in an overestimation or underestimation of the exposure [11, 12, 14, 54]. On the other hand, we have analyzed RA disease activity by means of three indexes (DAS28, DAS28-CRP and SDAI) in order to avoid the high heterogeneity reported when defining RA disease activity [10, 12, 34, 36]. This enabled us to establish the level of RA disease activity when two of the three used indexes agreed. Therefore, the results from this investigation provide a basis for further prospective interventional studies that could deepen our understanding of the association between periodontitis and RA.

## Conclusions

This study has demonstrated a significant association between RA and periodontitis, and more specifically between periodontitis severity and RA disease activity. These results suggest an independent relationship between severe periodontitis and RA in spite of common shared risk factors and other confounding factors affecting both diseases. Further well-designed prospective intervention studies are needed to elucidate how periodontitis may influence the development and progression of RA.

## Additional files

Additional file 1: Periodontal section. (DOC 25 kb)

Additional file 2: **Table S1.** Disease activity in early and established RA patients. **Table S2.** Linear regression model of clinical attachment level and RA disease activity. (DOC 59 kb)

Additional file 3: **Figure S1.** Relationship between RA disease activity assessed by different indexes and periodontitis severity. **Figure S2** Periodontal parameters in patients with RA in relation to their clinical activity. (DOC 399 kb)

## Abbreviations

ACPA: Anti-citrullinated peptide antibodies
Anti-CCP: Anti-cyclic citrullinated peptide
bDMARD: Biologic disease-modifying antirheumatic drug
BMI: Body mass index
BoP: Bleeding of probing
CAL: Clinical attachment level
CDAI: Clinical Disease Activity Index
CRP: C-reactive protein
DAS28: 28-joint Disease Activity Score
DMARD: Disease-modifying antirheumatic drug
ERA: Early rheumatoid arthritis
ESR: Erythrocyte sedimentation rate
HAQ: Health Assessment Questionnaire
OD: Odds ratio
PAD: Peptidil arginine deiminase
PI: Plaque index
PPD: Proving pocket depth
PSS-14: Perceived Stress Scale
RA: Rheumatoid arthritis
REC: Recession
RF: Rheumatoid factor
SD: Standard deviation
sDMARD: Synthetic disease-modifying antirheumatic drug
