# Supplementary material for: Association between severity of periodontitis and clinical activity in rheumatoid arthritis patients: a case–control study
Source: Arthritis Res Ther. 2019 Jan 18;21:27. doi: 10.1186/s13075-019-1808-z (PMC6339403; doi:10.1186/s13075-019-1808-z)
Supplement: Supplementary file 1 — Periodontal section. (DOC 25 kb) [file 13075_2019_1808_MOESM1_ESM.doc]

**Periodontal Section**

Periodontal Outcomes

Periodontitis is an oral chronic inflammatory disease that normally leads to periodontal attachment and bone destruction around teeth. The main cause of that destruction is periodontopathogenic bacteria. They are normally found around teeth in a biofilm structure that grow, even in a subgingival environment.

The primary outcome variable is clinical attachment level. However, there are other clinical variables that are also important in periodontitis. They are listed as follows:

- Clinical Attachment Level (CAL)

- Probing Pocket Depth (PPD)

- Recession (REC)

- Tooth Loss

- Plaque Index (PI)

- Bleeding On Probing (BoP)

Clinical Attachment Level(CAL)

It is measured as the distance from the cementoenamel junction (CEJ) to the bottom of the pocket. It could be calculated as the sum of recession and the probing pocket depth (REC+PPD).

Probing Pocket Depth (PPD)

It is the distance from the gingival margin to the bottom of the pocket.

Recession (REC)

It is defined as the distante from the CEJ to the gingival margin.

Plaque Index (PI) (25)

It is the assessment of soft and mineralized deposits around teeth. Third molars are excluded.

Four sites per tooth (buccal, lingual, mesial and distal) are taken. It ranges from 0 to 3:

0 = absence of deposits of plaque 1 = presence of plaque after passing the probe through the gingival margin 2 = visible plaque 3 = abundant plaque

Bleeding on Probing (BoP) (26)

It is registered after passing a periodontal probe in 10 seconds around the gingival margin. It ranges from 0 to 1.

0 = absence of bleeding and inflammation 1 = presence of bleeding and inflammation.

The number of positive sites is expressed as the percentage of sites with bleeding on probing.

25. Silness J, Löe H. Periodontal disease in pregnancy. II. Correlation between oral hygiene and periodontal condition. *Acta Odontol Scand* 1964;22:121–135.

26. Ainamo J, Bay I. Problems and proposals for recording gingivitis and plaque. *Int Dent J* 1975;25:229–235.
