# Supplementary material for: Association between severity of periodontitis and clinical activity in rheumatoid arthritis patients: a case–control study
Source: Arthritis Res Ther. 2019 Jan 18;21:27. doi: 10.1186/s13075-019-1808-z (PMC6339403; doi:10.1186/s13075-019-1808-z)
Supplement: Supplementary file 2 — Table S1. Disease activity in early and established RA patients. Table S2. Linear regression model of clinical attachment level and RA disease activity. (DOC 59 kb) [file 13075_2019_1808_MOESM2_ESM.doc]

**Table S1.** Disease Activity in Early and Established RA patients.

|  | **Early RA(<2 years)** | | **Established RA (>2 years)** | | **Total** | |  |
| --- | --- | --- | --- | --- | --- | --- | --- |
| **RA Disease Activity** | N | % | N | % | N | % |  |
| Remission | 4 | 11.43 | 34 | 22.37 | 38 | 20.32 |  |
| Low | 3 | 8.57 | 36 | 23,68 | 39 | 20.86 |  |
| Moderate | 17 | 48.57 | 68 | 44.74 | 85 | 45.45 |  |
| High | 11 | 31.43 | 14 | 9.21 | 25 | 13.37 |  |
| **Total** | 35 | 100 | 152 | 100 | 187 | 100 |  |
|  |  |  |  |  |  |  | p=0.001* |
| Data represent numbers and percentages.  RA: Rheumatoid Arthritis  * by Chi-square test. Kendall’s Tau b = 0.231 | | | | | | | |

**Table S2. Linear regression model of Clinical Attachment Level and RA disease activity.**

| **DAS28** | **β±SE** | 95%CI | p |
| --- | --- | --- | --- |
| Mean CAL | 0.24**±**0.08 | (0.09-0.40) | 0.002 |
| Gender (ref. to women) | -0.98**±**0.23 | (-1.44- -0.52) | <0.001 |
| Age | -0.02**±**0.01 | (-0.03- 0.00) | 0.091 |
| Smoking |  |  |  |
| Smokers | -0.14**±**0.25 | (-0.64-0.35) | 0.564 |
| Former Smokers | 0.14**±**0.23 | (-0.32-0.60) | 0.545 |
| Intercept | 3.81**±** 0.51 | (2.9-4.9) | <0.001 |
|  |  |  |  |
| **DAS28-CRP** | **β±SE** | 95%CI | p |
| Mean CAL | 0.19**±**0.02 | (0.04-0.33) | 0.011 |
| Gender (ref. to woman) | -0.62**±**0.21 | (-1.04- -0.20) | 0.004 |
| Age | -0.02**±**0.01 | (-0.03- 0.00) | 0.062 |
| Smoking |  |  |  |
| Smokers | -0.02**±**0.23 | (-0.47- 0.43) | 0.92 |
| Former Smokers | 0.17**±**0.21 | (-0.25- 0.59) | 0.421 |
| Intercept | 3.39**±** 0.47 | (2.47-4.31) | <0.001 |
| CAL: Clinical Attachment Level; CI: Confidence Interval; CRP: C-Reactive Protein; ESR: Erythrocyte Sedimentation Rate OR: Odds Ratio; RA: Rheumatoid Arthritis; SE: Standard Error. | | | |
