# Supplementary material for: Association between severity of periodontitis and clinical activity in rheumatoid arthritis patients: a case–control study
Source: Arthritis Res Ther. 2019 Jan 18;21:27. doi: 10.1186/s13075-019-1808-z (PMC6339403; doi:10.1186/s13075-019-1808-z)
Supplement: Supplementary file 3 — Figure S1. Relationship between RA disease activity assessed by different indexes and periodontitis severity. Figure S2 Periodontal parameters in patients with RA in relation to their clinical activity. (DOC 399 kb) [file 13075_2019_1808_MOESM3_ESM.doc]

Figure S1

**
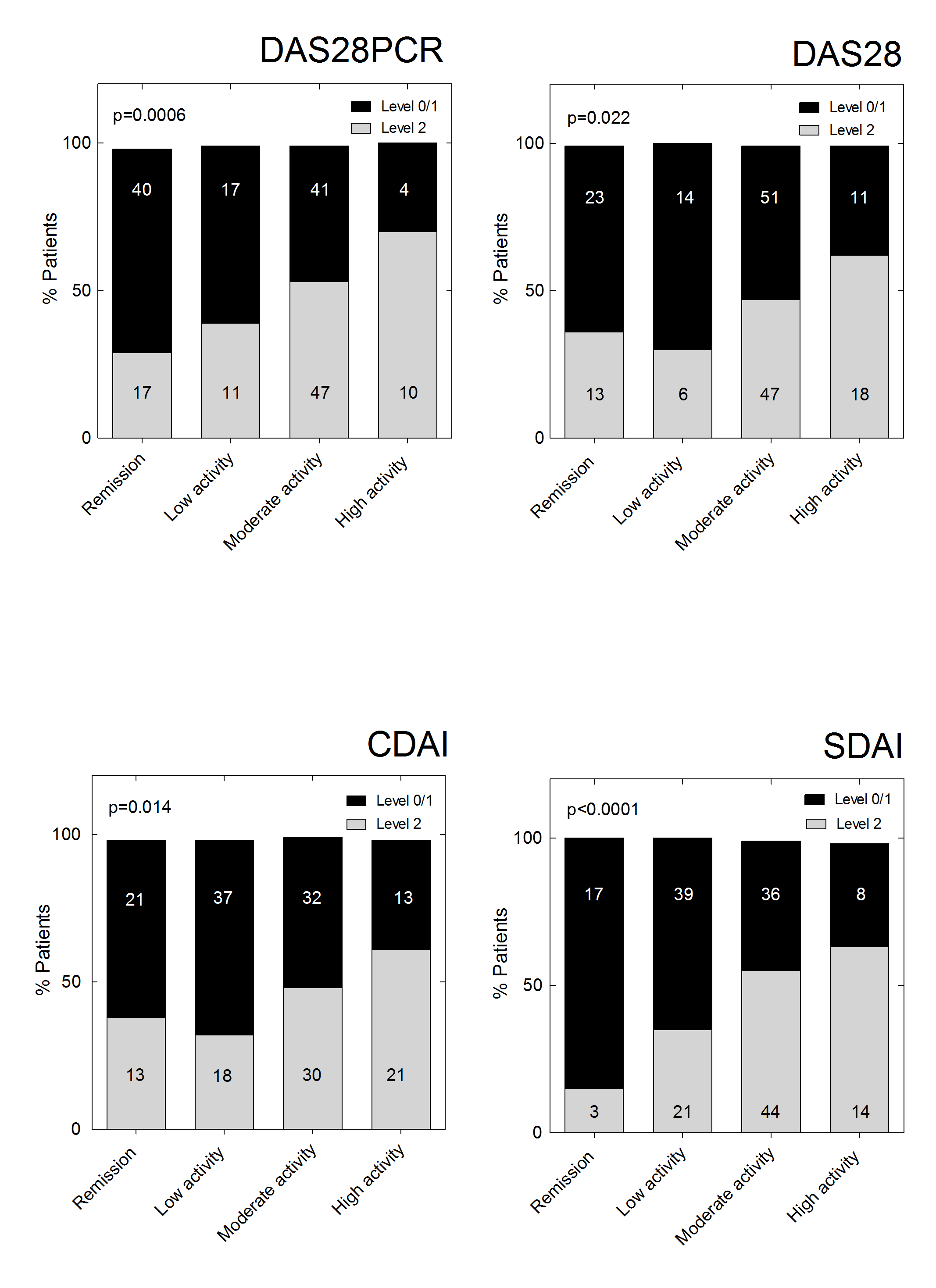
**

**Figure S1.** Relationship between RA disease activity assessed by different indexes and periodontitis severity. Stacked bar graphs showing the percentage of RA patients that presented none or mild (level 0/1) and severe (level 2) periodontitis with respect to RA disease activity assessed by DAS28-CRP, DAS-28, SDAI and CDAI and categorized as remission, low, moderate or high activity accordingly to threshold of each index. Numbers into columns represent the number of patients in each situation. The four graphs show relation between RA activity and periodontal state (all with p < 0.022 by Chi-Square test) and a linear increase in the level 2 ratio of periodontitis (Kendall’s Tau b of 0.237, 0.168, 0.169 and 0.271 for DAS28-CRP, DAS-28, CDAI and SDAI, respectively)

Figure S2


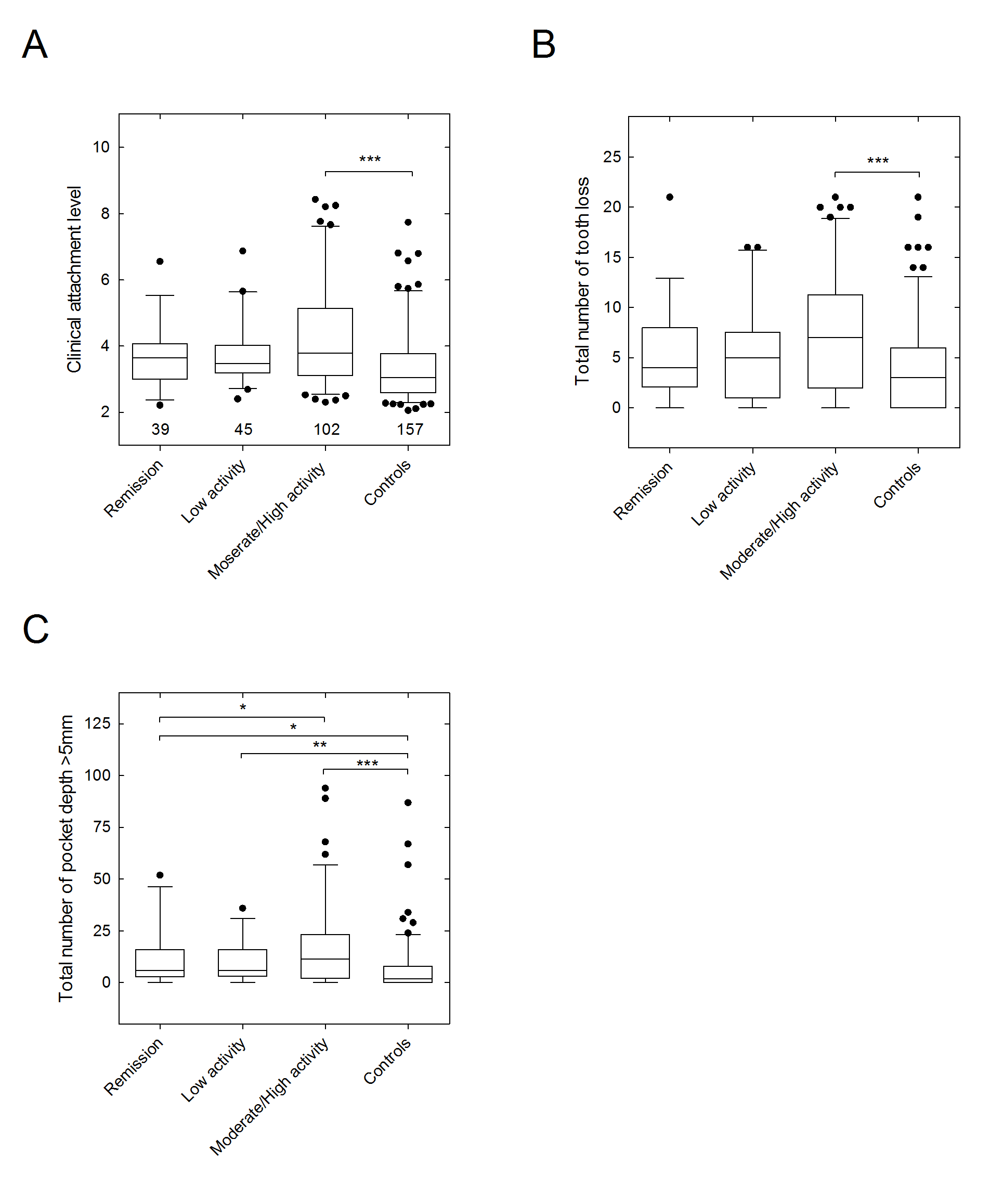


**Figure S2.** Periodontal parameters in patients with RA in relation to their clinical activity. Box plots showing the clinical attachment loss (A), tooth loss (B), and number of pockets ≥5 mm (C) in relation to the clinical activity in RA patients and controls. Data represent the 25th and 75th percentiles (boxes), the median (line within boxes), and the 95th and 5th percentiles (error bars). Numbers into the panel A represent the number of patients in each RA disease activity level. *p<0.05 and ***p<0.001 using for analysis one-way ANOVA with Dunnett´s multiple post-hoc comparisons.
